# Understanding the Return of Genomic Sequencing Results Process: Content Review of Participant Summary Letters in the eMERGE Research Network

**DOI:** 10.3390/jpm10020038

**Published:** 2020-05-13

**Authors:** John A. Lynch, Richard R. Sharp, Sharon A. Aufox, Sarah T. Bland, Carrie Blout, Deborah J. Bowen, Adam H. Buchanan, Colin Halverson, Margaret Harr, Scott J. Hebbring, Nora Henrikson, Christin Hoell, Ingrid A. Holm, Gail Jarvik, Iftikhar J. Kullo, David C. Kochan, Eric B. Larson, Amanda Lazzeri, Kathleen A. Leppig, Jill Madden, Maddalena Marasa, Melanie F. Myers, Josh Peterson, Cynthia A. Prows, Alanna Kulchak Rahm, James Ralston, Hila Milo Rasouly, Aaron Scrol, Maureen E. Smith, Amy Sturm, Kelsey Stuttgen, Georgia Wiesner, Marc S. Williams, Julia Wynn, Janet L. Williams

**Affiliations:** 1 Department of Communication, University of Cincinnati, Cincinnati, OH 45220, USA; 2 Biomedical Ethics, Mayo Clinic, Rochester, MN 55902, USA; sharp.richard@mayo.edu; 3 Center for Genomic Medicine, Feinberg School of Medicine, Northwestern University, Chicago, IL 60611, USA; s-aufox@northwestern.edu (S.A.A.); christin.hoell@northwestern.edu (C.H.); 4 Department of Biomedical Informatics, Vanderbilt University Medical Center, Nashville, TN 37232, USA; sarah.bland@vumc.org (S.T.B.); josh.peterson@vumc.org (J.P.); 5 Harvard Pilgrim Health Care Institute, Boston, MA 02115, USA; cblout@bwh.harvard.edu; 6 Department of Bioethics and Humanities, School of Medicine, University of Washington, Seattle, WA 98195, USA; dbowen@uw.edu; 7 Genomic Medicine Institute, Geisinger, Danville, PA 17822, USA; ahbuchanan@geisinger.edu (A.H.B.); allazzeri@geisinger.edu (A.L.); akrahm@geisinger.edu (A.K.R.); asturm@geisinger.edu (A.S.); mswilliams1@geisinger.edu (M.S.W.); jlwilliams3@geisinger.edu (J.L.W.); 8 School of Medicine, Indiana University-Purdue University, Indianapolis, IN 46202, USA; chalver@iu.edu; 9 Center for Applied Genomics, Children’s Hospital of Pennsylvania, Philadelphia, PA 19104, USA; HARRM@email.chop.edu; 10 Marshfield Clinic Research Institute, Marshfield, WI 54449, USA; hebbring.scott@marshfieldresearch.org; 11 Kaiser Permanente Washington Health Research Institute, Kaiser Permanente of Washington, Seattle, WA 98101, USA; nora.b.henrikson@kp.org (N.H.); Eric.B.Larson@kp.org (E.B.L.); James.D.Ralston@kp.org (J.R.); aaron.scrol@kp.org (A.S.); 12 Department of Health Services, School of Public Health, University of Washington, Seattle, WA 98195, USA; 13 Department of Pediatrics, Harvard Medical School, Boston, MA 02115, USA; Ingrid.Holm@childrens.harvard.edu; 14 Division of Genetics and Genomics, Boston Children’s Hospital, Boston, MA 02115, USA; jill.madden@childrens.harvard.edu; 15 Departments of Medicine (Medical Genetics) and Genome Sciences, University of Washington, Seattle, WA 98195, USA; gjarvik@medicine.washington.edu; 16 Department of Cardiovascular Medicine, Mayo Clinic, Rochester, MN 55902, USA; kullo.iftikhar@mayo.edu (I.J.K.); Kochan.David@mayo.edu (D.C.K.); stuttgen.kelsey@mayo.edu (K.S.); 17 Division of General Internal Medicine, University of Washington, Seattle, WA 98195, USA; 18 Genetic Services, Kaiser Permanente of Washington, Seattle, WA 98101, USA; Kathleen.A.Leppig@kp.org; 19 University of Washington Biomedical and Health Informatics, Seattle, WA 98195, USA; 20 Department of Medicine, Division of Nephrology, Columbia University Irving Medical Center, New York, NY 10032, USA; mm4440@cumc.columbia.edu (M.M.); hm2673@cumc.columbia.edu (H.M.R.); 21 College of Medicine, University of Cincinnati, Cincinnati, OH 45220, USA; Melanie.Myers@cchmc.org; 22 Department of Pediatrics, Cincinnati Children’s Hospital Medical Center, University of Cincinnati, Cincinnati, OH 45229, USA; cindy.prows@cchmc.org; 23 Department of Medicine, Vanderbilt University Medical Center, Nashville, TN 37232, USA; Georgia.wiesner@vumc.org; 24 Department of Medicine, Feinberg School of Medicine, Northwestern University, Chicago, IL 60611, USA; m-smith6@northwestern.edu; 25 Vanderbilt Clinical and Translational Hereditary Cancer Program, Vanderbilt-Ingram Cancer Center, Vanderbilt University Medical Center, Nashville, TN 37232, USA; 26 Department of Pediatrics, Columbia University Irving Medical Center, New York, NY 10027, USA; jw2500@cumc.columbia.edu

**Keywords:** genomic medicine, genetic testing, return of results, patient communication, written communication

## Abstract

A challenge in returning genomic test results to research participants is how best to communicate complex and clinically nuanced findings to participants in a manner that is scalable to the large numbers of participants enrolled. The purpose of this study was to examine the features of genetic results letters produced at each Electronic Medical Records and Genomics (eMERGE3) Network site to assess their readability and content. Letters were collected from each site, and a qualitative analysis of letter content and a quantitative analysis of readability statistics were performed. Because letters were produced independently at each eMERGE site, significant heterogeneity in readability and content was found. The content of letters varied widely from a baseline of notifying participants that results existed to more detailed information about positive or negative results, as well as materials for sharing with family members. Most letters were significantly above the Centers for Disease Control-suggested reading level for health communication. While continued effort should be applied to make letters easier to understand, the ongoing challenge of explaining complex genomic information, the implications of negative test results, and the uncertainty that comes with some types of test and result makes simplifying letter text challenging.

## 1. Introduction

There is a growing consensus that the results of genomic testing ought to be returned to research participants, especially when those results may be viewed as useful or may have implications for health [1,2,3,4]. Multiple projects have started offering results from genome sequencing, exome or targeted sequencing, and other multi-gene results to research participants [5,6]. Nonetheless, numerous questions remain regarding which results to offer and in what manner. These are topics of much interest in the context of several large National Institutes of Health (NIH) consortia, including Electronic Medical Records and Genomics (eMERGE), Implementing Genomics in Practice (IGNITE), Clinical Sequencing Evidence-generating Research (CSER), and others.

In traditional clinical genetics practice, visits are typically followed by a detailed clinic summary letter shared with the patient [7]. Clinic letters, in general, serve the purpose of documenting relevant facts for patients and their caregivers by presenting information in a way that promotes understanding [8,9]. Anecdotally, providers have reported that these letters often serve the additional purpose of briefly summarizing complex genetic test results and suggesting clinical support resources that aid primary care providers and other non-genetic specialists who may be involved in the patient’s care. Issues of readability (number of words, length of sentences and paragraphs, complexity, use of jargon, use of passive sentences, etc.) have been identified as key concerns in improving patient understanding of clinic letters [8,9,10]. The complexity of some genetic results, including variants of unknown significance and uninformative negative results, can exacerbate the general issues of health communication coupled with low health literacy [11,12].

An ongoing challenge in returning genomic test results to research participants is how best to communicate complex and clinically nuanced findings to the participants and their primary healthcare providers (PCP) in a manner that is scalable to the large numbers of research participants enrolled. Writing effective summary letters takes time, up to 20–30 min per letter [13]. It is unclear whether providing nuanced, participant-tailored summary letters, given the increasing array of genomic tests being offered, is feasible in settings with very large numbers of research participants or for research studies that make use of stored biological materials, where direct contact between participants and research staff may be minimal. Such an investment may be costly or may require genetic counseling resources that are not readily available. Directing resources toward these efforts also might not be wise since we know very little about the extent to which participants understand these communications or make use of them.

Additionally, there has been little standardization of the methods for communicating sequencing results to participants. Some research groups have developed patient-friendly genomic test reports [14,15,16,17]. Such communication may also be supported by ancillary materials such as educational brochures and websites or opportunities to follow up with a genetics professional in person. These patient- and participant-friendly test reports and the use of ancillary materials have not yet been widely adopted in clinical practice or in research settings, although their use has been identified as one way to improve patient and research participant engagement and understanding [15].

Research networks, like eMERGE, that are returning genomic results must adapt existing communication practices in response to these practical constraints, potentially sending genomic test reports to participants through their electronic health records (EHRs) or providing highly condensed summary letters notifying participants of new findings and encouraging them to connect to a member of the research team to learn more. To date, research studies examining the real-world communication of genomic results have been lacking, especially studies that examine the content and structure of letters communicating genomic test results. The eMERGE Network, comprised of ten research sites, all charged with returning research results to their participants, provided the opportunity to study what researchers developed as written communications to participants of genomic research results.

The purpose of this study was to examine features of genetic results letters (defined as all written communication provided by mail, email, and electronic health records) independently produced at each eMERGE site to assess their readability and content, their management of uncertainty, and the utilization of interpersonal and coping elements. Our aim was to characterize emerging practices for communicating research genomic test results through written communications and inform future efforts to develop scalable, patient-centered approaches to reporting research genomic test results.

## 2. Materials and Methods

### 2.1. Study Setting

The eMERGE Network consists of sites that have established biobanks linked to electronic health records (EHRs) [18,19]. In its third funding round, eMERGE 3, central clinical genetics laboratories sequenced DNA from approximately 22,500 participants across the 10 eMERGE Clinical sites using a next generation sequencing panel of ~100 genes with high penetrance and strong evidence for association with a medically actionable condition, including the genes on the ACMG list, as well as actionable findings at 14 single nucleotide variants [20]. All sites returned positive genomic results (pathogenic or likely pathogenic variant in a gene on the panel) to participants and their PCP and integrated the result into the EHRs for clinical care. All written communications to participants were reviewed and authorized by the Institutional Review Board at each site. The first author (JAL) collected the participant letter templates from each site that were used in 2018 and 2019 and clarified the various time points at which the letters were made available to participants, specifically when the letters were provided in relation to other key events such as the uploading of genomic results into the EHR, the communication of the results to the participant’s PCP, and any participant contact with a medical geneticist or genetic counselor. The content of any ancillary educational materials was discussed by individual sites as appropriate.

### 2.2. Study Analysis

The research team conducted both a qualitative and quantitative analysis of the participant letters. The research team conducted a qualitative analysis of the letters. Codes were developed through an iterative process that involved reviews of the existing literature on the return of research results and letters in clinical genomic practice, alongside a review and close reading of the participant letters. Six coding categories were identified: letter type (notification of available results, positive results, negative results, family communication), genomic terminology and definitions, individual risk, family risk, epistemic stance (i.e., the certainty or uncertainty of knowledge expressed in factual statements), and affective stance (i.e., positive or negative emotional statements reflecting interpersonal content and coping mechanisms). Coding was conducted by the first and last authors (JAL and JLW), who resolved all coding disagreements through consensus. To ensure that coding reflected the experiences of writing and using the letters, coded documents and a copy of the coding scheme were returned to a representative from each site who reviewed the coding in coordination with the first author, made suggested changes, and provided final approval of the site coding.

The research team then used investigator triangulation to analyze the results of the coding [21]. Specifically, they reviewed the aggregate coding results to identify potential conclusions about participant letters. These initial conclusions were refined through the discussion of site experiences and a review of the existing literature on patient/participant communication.

Descriptive statistics were used to summarize measures of readability. Quantitative measures included word count, the number of words per sentence, the number of sentences per paragraph, the percentage of sentences that used the passive voice, the number of URLs to additional resources, and the Flesch–Kincaid reading grade level. To account for the use of definitions and explanations of medical jargon, each appearance of the defined term was replaced by the word “cat” before the Flesch–Kincaid reading grade level was assessed [14].

## 3. Results

### 3.1. Communication Methods

**Figure 1:**
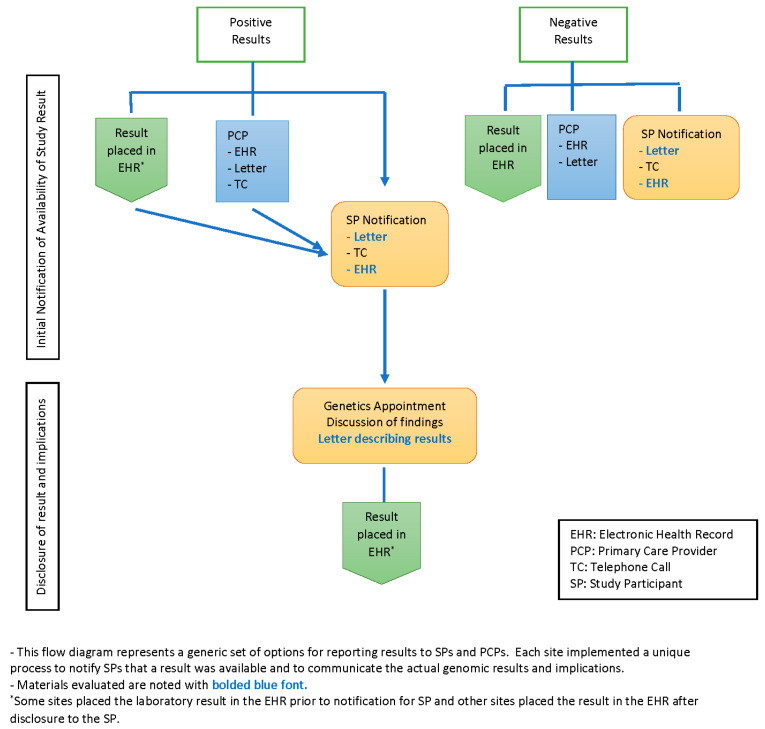
Flow diagram of return of result sequences for positive and negative results.

All sites used more than one medium for communicating medically actionable research results to participants, including letters, telephone contact and the patient portal of the EHR. In addition, sites used combinations of all three media at varying points and for varying purposes during the research return of results (ROR) process; see Figure 1. Three sites first notified participants that a result was available by a brief phone call; full research findings were then returned in a scheduled phone or in-person visit. Two sites notified participants that a research result was available first by letter. Two sites sent notification letters in tandem with a telephone call to participants, and three sites sent a letter to the participant and the PCP in tandem.

In addition to the notification letter, several sites sent participants a letter with the actual result and varying amounts of information about the condition associated with the finding. The content resembled a typical genetics visit summary that is usually sent following a traditional clinical genetics consultation, though two sites employed this letter prior to the scheduled full disclosure visit. Seven sites sent a participant summary letter after an in-person and/or telephone counseling visit. One site had the participants choose at the start of the study how they would like to receive their results: by phone, in person or through the receipt of a secure email, with results returned according to the participants’ choice regardless of the type of result.

For negative results, three sites notified participants of negative results by letter in tandem with a letter to the PCP; five sites sent notifications via the patient portal and/or sent a participant letter; and one site, with two cohorts, sent a letter in tandem with an additional telephone call for one of their cohorts. Two sites did not return negative results and so had no letters to review.

### 3.2. Letter Type

Ten eMERGE sites provided 43 letters that were analyzed (Table 1). The number of letters per site ranged from one to 17 letters, with a median of three letters per site. Thirty-eight letters were directed at the individual participant or the parents of pediatric participants. Of those, twenty-four letters were involved the return of positive (pathogenic or likely pathogenic variant) results associated with a known genetic condition, seven letters reported negative genomic sequence findings, five notified participants a result was available in the EHR or through the research team, one letter returned the finding of a variant of uncertain significance, and one letter written to the individual participant encouraged them to share results with their family. Four letters were created for the families of individuals who received genomic results, and one letter was designed for family members of a participant who had passed away prior to the return of the genomic test results.

### 3.3. Genomic Terminology and Definitions

Letters employed a range of key terms from genetics and genomics (Table 2). Some sites provided definitions to clarify the meaning of terms and make letters easier to understand, with one site including a glossary at the end of all positive results letters.

All sites referenced “genetic” tests or testing in one or more letters. Letters from nine sites mentioned that the test examined “genes”, and seven sites had all letters referencing “genes”. Only five letters across all sites did not use the word “gene”. Four of these letters were notifications of available results, and the last letter was a cover letter accompanying a notice participants could provide to family members that discussed a participant’s positive test results. Only four sites provided a definition for “gene” in their letters.

The term “variant” was accompanied by many different adjectives (e.g., “actionable”, “pathogenic” or “disease-causing”). Thirty-one of 43 (72%) letters from nine sites used “variant”, and 24 of those 43 (56%) letters from five sites defined the term. Definitions ranged from one-word substitutions to sentence-length descriptions. Eight letters did not use “variant”: two reported negative results, two were communications to family members, three reminded participants of prior telephone conversations about results or directed participants to call about their results, and one letter informed participants that their PCP had been notified and would contact them. Three letters from three sites used the term “mutation”. Two of the three letters provided brief definitions of the term mutations: “changes” in one and “pathogenic variant” in the other.

Sixteen letters from four sites used the term “dominant” or “autosomal dominant”. All but one of those letters offered definitions of the term. Definitions included sentence- or paragraph-length descriptions, and some letters provided images illustrating autosomal dominant patterns of inheritance. Pediatric sites returned autosomal recessive disease-related genes, and these sites used and defined the term “autosomal recessive”. Those definitions were text-only.

### 3.4. Individual Risk

Thirty-seven of the 43 (86%) letters across nine sites included qualitative or quantitative descriptions of individual risk. Six letters from four sites did not include any description of risk: one letter informed participants that their PCP would contact them; one letter from one site reported negative results; two letters from two sites informed participants about positive results; and two letters from one site reminded participants of a prior phone call and reminded them information is available in their online medical record. Thirty-one letters (72%) from nine sites offered qualitative descriptions of risk. The language used to indicate risk varied: “at risk”, “higher risk”, “increased risk” and “significantly increased risk” were all used. Occasionally, letters used terms of increasing risk severity; e.g., “risk” became “increased risk” and “significantly increased risk” over the length of the letter, but this pattern was sporadic and was not unique to any one site or type of letter.

Eleven letters (26%) from four sites provided quantitative risk information. These quantitative indicators of risk were prefaced by qualitative descriptors, most commonly “at risk” or “increased risk”. Ten of the letters focused on cancer risks, and eight of those letters compared cancer risks for those with the gene variant being discussed with the cancer risks for a population without the gene variant.

### 3.5. Family Risk

Positive results letters and letters to family also provided qualitative and quantitative descriptions of family disease risk and genetic inheritance risks. Thirteen of 30 (43%) letters from four sites mention family disease risk. Of those 13 letters, five letters from three sites say family members may be at “increased risk”; two letters from one site said family members “may have the same risk” as the individuals tested, shortly after describing the quantitative risk increases for the individual tested. The remaining six letters from these four sites indicated that family members may be “at risk” without further description.

Some letters also stated that family members are at a genetic risk, meaning they might have also inherited the condition. Eighteen of 30 (60%) letters from four sites indicated that first-degree relatives have a 50% chance of inheriting or having the variant. Six of 30 (20%) letters from three sites mentioned carrier status for recessive conditions. See Box 1.

Box 1 **Examples: Familial risk language.**

“Because we share genes with family members, some of your relatives may have the same genetic risk. You should share this result with relatives so they are aware that they could be at-risk for disease”.

“Your parents, children, brothers and sisters are at 50% risk for the same genetic change. A simple “yes/no” blood test will allow them to better understand their risk”.

“Share a copy of the enclosed letter with each of your living parents, children, siblings. Follow-up is EXTREMELY important to guide healthcare not only for them but also for their family”.

### 3.6. Epistemic Stance

Letters offered both certain and uncertain epistemic stances (the certainty or uncertainty of knowledge expressed in factual statements). When the epistemic stance was certain, statements typically were direct, simple sentences; see Box 2.

Box 2 **Examples: epistemic statements of certainty.**

You have positive results for the following genes: [gene name].

A single likely pathogenic variant in the [gene name] gene was identified.

A genetic change [Variant c. p.] was identified in one out of two copies of your [Gene Name] gene.

Your genetic testing identified a [variant] [pathogenic/likely pathogenic] variant in the [gene name] gene.

The exact gene change that was found is [c.xxx > A (p.AxxxLys)] in the [gene name] gene.

All letters reaffirmed that participants had had genetic testing done; letters to individuals reminded them the test had been conducted, while letters to family members stated that a relative was in a study that included genetic testing. All letters reporting positive results affirmed that a genetic variant was found that increased one’s risk of a health condition, and all letters affirmed that results would be placed in their electronic health record. In negative results letters, qualifying statements were common, although two sites used language indicating certainty when reporting the limits of negative results. Instead of saying that participants “may have a higher risk”, the letters made statements that negative results do not eliminate the risk of developing a disease.

Letters often included qualifying statements that tempered statements that had been previously made with higher levels of epistemic confidence (see Box 3).

Box 3 **Examples: qualifying statements in summary letters.**

“Receiving a genetic result does NOT mean that you have a health problem. This result means that you are likely at higher risk to have a health problem in the future”.

“Research findings are not the same as clinical tests done during routine clinical care. Your doctor may want to repeat the research test using a certified clinical laboratory to be certain that the result is correct”.

“Individuals with variants in this gene have an increased risk for a disease called [disease name] that may result in an increased risk for [health risks]”.

Not all letters provided statements using uncertainty language, but uncertainty language was used in all letter types. Only 22 (51%) letters from nine sites offered variations of the statement that participants “may have a higher risk” for disease but that it was not certain they had or would develop a disease with a genetic component.

Five (12%) letters from three sites stated that tests did not consider all genetic variants, and four (9%) letters from three sites indicated that researchers could find new risk factors in the future. Five (12%) letters from four sites said family members could have the same gene variant as the participant but that they could not confirm that without testing for family members, and three (7%) letters from two sites stated that research findings are not the same as clinical findings.

In addition to issues regarding testing and family risk, some letters offered an epistemic stance on the certainty of science (see Box 4). Sixteen of 43 letters (37%) from seven sites referenced scientific progress in order to warn participants that the meaning of test results could change as knowledge continues to develop. Finally, a small subset of letters offered uncertain epistemic stances about practical issues; five (12%) letters from three sites warned participants that recontact by the research site was not guaranteed if new results were found or new interpretations of existing results were made.

Box 4 **Examples: epistemic stance on the evolving state of science.**

“Our knowledge of the meaning of genetic variants is evolving. We may send an update to you and to your doctor”.

“Future research may find more disease-causing variants”.

“What we know about genetics is always changing. If our understanding of your results changes within the course of the study, we will contact you”.

### 3.7. Affective Stance

Letters reporting both positive and negative results generally provided a positive affective stance—emotion statements that provide interpersonal context for the letter and identify coping behaviors (see Box 5). Seven letters from five sites thanked participants for being in the eMERGE study. Most letters encouraged participants to engage in proactive actions. The most common affective stance encouraged participants to believe they can improve their health or effectively manage any health condition indicated by the genetic test, and it was found in 27 of 43 (63%) letters across eight sites. Similarly, 24 (56%) letters for positive and negative results across six sites encouraged participants to contact a genetic counselor. However, the statements about contacting a genetic counselor to talk about results were split per two conditions; the majority (19 letters) indicated that one should contact a genetic counselor if one were concerned or had questions about the results received, but five letters encouraged participants to contact a genetic counselor every few years to see whether recommendations for screening or treatment related to their genetic test result had changed. The next most common stance was to encourage participants to share results with family, found in 20 (47%) letters across four sites, followed by encouragement for participants to share results with their PCP (10 letters, seven sites).

Box 5 **Examples: positive affective stance.**

“We would like to thank you for your involvement and support in this research project”.

“Please keep this letter with the test report, and share them with both your doctors and family members”.

“Please speak with a genetic counselor to discuss if additional testing is recommended for you”.

“You should share this result with relatives so they are aware that they could be at-risk for disease”.

Very few letters had negative affective stances. One letter noted that the information being shared in the letter might be troubling for participants. Some letters emphasized that participants needed to quit smoking, and a letter from one site warned participants to not use participation in a research study as a substitute for clinical genetic testing. Only four letters offered no positive or negative affective stance.

### 3.8. Measures of Readability

Letters varied widely in length, ranging from 97 words to 2458 words, with a mean of 660 words. Across all sites, a wide range existed for all readability statistics, including the number of words, words per sentence, sentences per paragraph, the percentage of passive sentences, and the Flesch–Kincaid grade level (Table 3). The Centers for Disease Control (CDC) recommends that health communications should read at the seventh grade level [22]. The percentage of passive sentences in each site’s letters ranged from 10% to 27%, with an mean of 20.12%, and the Flesch–Kincaid grade level ranged from 7.67 to 13.95 (i.e., seventh grade to a freshman/sophomore in college), with a mean of 10.33 (i.e., a sophomore in high school). The use of definitions in letters lowered the Flesch–Kincaid grade level by 0.5 grades, indicating that definitions helped simplify some material and make it potentially easier to understand. The amount of external material, represented by URLs, ranged from 0 to 13. The two letters from one site with 13 URLs were outliers. When these were removed, the number of URLs ranged from 0 to 5. URLs appeared both in physically mailed letters and those sent electronically. Most letters provided no links to external material, but when URLs were used, they provided links to a range of materials, including resources for finding genetic counselors, information on genetics, information on disease condition, and links to resources (videos, webpages, etc.) unique to individual sites. A similarly wide range existed for all readability statistics across letter types (Table 4).

One site achieved a Flesch–Kincaid level of about sixth grade in one communication using pictorial vignettes to explain negative results, and another site achieved a Flesch–Kincaid of seventh grade in their notification letter for negative results. One site achieved Flesch–Kincaid measures of about eighth grade in their positive results letter using a question and answer format. Notification letters had the highest average words per sentence and the highest Flesch–Kincaid grade level of all letter types, and one site with two notification letters measured each at a sophomore in college level.

## 4. Discussion

This study analyzed written communications explaining genetic test results to participants in the eMERGE network. The implementation procedure for informing participants of their results varied across sites, from a baseline of notifying participants that results existed to more detailed information about positive or negative results, as well as materials for sharing with family members. The letters operated in a network of additional communication, including telephonic and in-person contact with researchers, information in EHRs and patient portals, and contact with PCPs, as well as additional print resources and links to URLs.

The lack of harmonization of the return of results strategies in eMERGE can be seen as a significant limitation in this study, as well as in other aspects of measuring the eMERGE network outcomes. However, it also provides a real-world look at implementation strategies in returning genomic sequencing results. The wide variation in communication strategies and content across eMERGE sites suggests that the issue of how best to report genomic test results has not been resolved. Despite the variation in eMERGE, our analysis highlights several places with a moderately high level of agreement in approach and/or content. It is important to note, however, that high agreement does not imply that what we found is the most effective approach to communication; neither should it indicate that there is no room for improvement throughout the process. There may have been agreement simply because it reflects common communication strategies in clinical practice. Similarly, the variation we observed could reflect particular issues encountered by individual network sites, such as specific Institutional Review Board requirements, and thus changes may be iterative attempts to improve upon traditional procedures. It is also important to note that there were places marked by considerable disagreement, which may provide opportunities for improvement.

Almost all of the sites used the term “gene variant” rather than “mutation” when discussing genetic test results, which is the language supported by existing ACMG guidelines [23]. The word “mutation” was used by some sites, and while those usages conformed to the technical definition of the term (i.e., that a genetic sequence deviated from the standard sequence and is not necessarily deleterious) [24], prior research shows that lay audiences react negatively to the word “mutation” [25,26]. The use of the term “variant” led to letters that provided participants the terms preferred by the ACMG while avoiding language shown to produce negative affective outcomes. The modification of “variant” with different adjectives has not been studied. Future research on how participants understand the terms “actionable variant”, “disease-causing variant”, “pathogenic variant” and “genetic variant” is needed in order to develop guidance on the preferred language for communicating genetic test results to participants (or patients) that will enhance understanding and avoid unnecessary negative reactions.

Letters included a range of qualitative and quantitative descriptions for both individual and family risks. Qualitative descriptions avoided extreme or emotional language, emphasizing, at most, “higher risk”. For some letters, quantitative descriptions of risk were blended with language of individuals and families being “at risk” or “at increased risk”. The use of qualitative risk in results letters for multiple conditions or conditions where risk is not well-defined and the use of quantitative risk descriptions in cancer genetics comports with existing best practice [27,28,29]. As knowledge of the variant penetrance and relationship with disease risk develops, letters should incorporate appropriate quantitative descriptions of disease risk.

A review of the epistemic and affective stances of the letters identified several common elements regardless of letter purpose. They included a reminder of study participation and/or an expression of thanks for participation; a confirmation that genetic testing was completed, that participants consented to receive results, and that a result was found; encouragement to speak with a genetic counselor and/or their PCP; a description of the relevance of their result for family members; and the contact information of the study team. Sites that did not include these elements may wish to consider adding the missing content to their participant communications in future studies, as this seems to represent a de facto standard of care.

Sites produced a range of different letter types with different purposes. Some sites sent participants only a notification letter, while others sent notification letters, letters summarizing results and letters for family members. The purpose of each communication was anticipated to influence the readability and qualitative analysis of the letters, with notification letters expected to have the best readability measures and be the easiest to understand. However, notification letters had some of the lowest readability measures. Sites might have spent less effort creating notification letters, in contrast to positive and negative results letters, leading to notification letters that were less readable. Anecdotally, some researchers described difficulty in crafting a notification letter that would convey enough information to encourage a participant to follow-up with a visit but not disclose the actual result before meeting with the participant. Summary letters for positive results were expected to have the most jargon and potentially present lower readability measures, but those letters were more readable than notification letters. This may not be surprising as this type of letter is most similar to clinical visit summaries, which genetics providers have significant experience in crafting. Overall, the analysis revealed that the site, rather than the purpose of the letter, had a greater influence on measures of readability and understanding. Sites that achieved a lower grade level, and thus were perhaps more effective in communicating results, provided definitions of terms. Definitions helped to lower reading levels in all letters in which they appeared despite the fact that the number of words increased. This approach could be incorporated into future communication plans, to enhance the readability of written communications. Creating a standard term glossary for patients informed by examples such as the NHGRI’s talking glossary of genetic terms (https://www.genome.gov/genetics-glossary) could be useful.

The need to provide information that is both portable and easy to understand remains an ongoing challenge in medical genetics. Prior qualitative studies document participants’ desire for a permanent record of genetic findings (a) that is easy to understand, (b) outlines what they need to do to use the genetic results to improve their healthcare, and (c) that is portable to the various different healthcare settings that they may visit and that use different EHR systems [14,15,16]. While our study does not provide definitive evidence for the best practices in letter writing, it does identify several potential elements to be considered for effective written communication to participants in large-scale genomic research (Table 5). Our findings identify potential letter content as well as suggestions for the letter writing process, but continued effort should be applied to make letters simpler or easier to understand. One of the most challenging issues is the readability of letters. Possible strategies to address readability include using a question-and-answer format, avoiding overly technical language or terms, adding definitions as necessary, and providing simple, straightforward options for next steps and for contacting individuals.

Further qualitative studies should explore whether participants read the letters, as well as what participants comprehend from the results and notification letters. Previous studies have designed letters with the input of patient/participants [14,15]. Therefore, it will be important for researchers to obtain the input of their participants while developing results communications. Additionally, it will be important to study alternative methods of presenting results including the use of images to define terms, the use of visual aids to explain quantitative risk measures, and additional ways of describing genetic variation. Current means of assessing readability, like the Flesch–Kincaid grade reading level, cannot assess the readability of images. Engaging health literacy experts to review materials is an additional resource. Such focused study could add to the needed repertoire of resources to address concerns of effective health communication and low health literacy.

The current study’s pragmatic design, which was dictated by network and individual site resources, could act as a limitation of the results. In addition, this research was conducted with populations that were largely white, northern Europeans, who received care at large academic health centers, representing a lack of participant diversity. For all but one site, materials were only available in English. Health literacy was not routinely assessed prior to enrollment. Future research should seek to engage more diverse patient communities to assess their needs in relation to participant-centered communications. In addition, while the heterogeneity of procedures across sites provided significant variation to analyze, a review of the letters did not lend itself well to a direct comparison across sites and within communication modalities. Another limitation was that the importance of the communication content was recognized after implementation of the return of the results process had begun. Consequently, the viewpoint of participants regarding the style, content, language and usability of the information was not incorporated into the communication strategies developed by each site.

## 5. Conclusions

This review of written communications of genomic sequencing results, which was developed as part of a large NHGRI-funded research consortium, identified areas for improvement that may guide future large research projects that intend to return genomic sequencing results to participants. Written materials developed to communicate genomic sequence results in this large research network were found to be overly complex and to exceed recommended readability measures. Written materials also had a range of common elements, like the use of “variant” to describe genetic findings, as well as epistemic and interpersonal elements that can facilitate communication. Examples of appropriate readability utilized well-recognized aids including the incorporation of definitions or the addition of a glossary for technical terms. Written materials will be improved by taking advantage of known strategies to improve written communication, such as using short sentences, including definitions of technical language, being aware of how certainty is framed, and creating positive affective content in the letters. Several resources are available to guide this process (e.g., PRISM [30] and AHRQ [31]), as well as experts in many healthcare systems. Researchers would be well served to utilize these resources. Greater investment at the outset of the research protocol, with participant involvement up front, will improve written communication to participants in future genomic sequencing projects.

## Figures and Tables

**Table 1:** Letter Types.

Site	Notification	Positive Results	Negative Results	Family	Total
1	0	3	0	0	3
2	0	1	1	1	3
3	0	2	0	0	2
4	0	2	2	0	4
5	2	2	0	1	5
6	1	0	0	0	1
7	1	1	1	0	3
8	0	1	1	0	2
9	2	0	1	0	3
10	0	13	1	3	17
Total	6	25	7	5	43

**Table 2:** Frequency of appearance and definition of key terms.

Term	Alternate Phrasings	Number of Sites
Gene	genetic; medically significant genetic finding	9 sites used; 4 sites defined
Variant	actionable variant; disease-causing variant; genetic alteration; genetic change; genetic variant; likely pathogenic variant; mutation; non-pathogenic variant; pathogenic variant;	9 sites used; 5 sites defined
Dominant	autosomal dominant	4 sites used; 4 sites defined
Recessive	autosomal recessive	2 sites used; 2 sites defined

**Table 3:** Readability statistics by site.

Site	Letters	Words	Words/Sentence	Sentence/Paragraph	Passive Sentences	Flesch–Kincaid Grade Level
1	3	232.67	21.73	3.77	27.00%	11.53
2	3	328.67	15.00	1.97	25.67%	8.47
3	2	1482.50	17.65	3.00	24.50%	12.25
4	4 *	443.67	14.97	2.30	23.25%	9.23
5	5	1050.20	16.14	2.18	10.00%	9.76
6	1	203.00	20.30	3.30	20.00%	12.70
7	3	396.33	15.07	2.67	12.67%	7.67
8	2	277.50	24.20	2.15	22.50%	13.95
9	3	231.33	19.07	2.67	26.00%	12.53
10	17	815.18	15.64	2.85	19.65%	10.15
Total	43 *	660.24	16.86	2.69	20.12%	10.33

* One letter was a macro that included information for multiple conditions. It was excluded from analysis for number of words, words/sentence and sentences/paragraph.

**Table 4:** Readability statistics by letter type.

Letter Type	Letters	Words	Words/Sentence	Sentence/Paragraph	Passive Sentences	Flesch–Kincaid Grade Level
Notification	6	250.17	18.20	2.80	15.33%	11.13
Positive/VUS	24	932.75	16.83	2.64	21.44%	10.32
Negative	7	285.00	16.37	2.27	20.57%	10.02
Family	5	369.60	16.08	3.40	18.60%	9.80
Total	42 *	660.24	16.86	2.69	20.12%	10.33

* One letter was a macro that included information for multiple conditions. It was excluded from analysis for number of words, words/sentence, and sentences/paragraph.

**Table 5:** Potential letter component and writing process standards from the Electronic Medical Records and Genomics (eMERGE) experience.

Potential letter elements Use of gene “variant” language to describe test results Glossary of technical and genetic terminology Thank you for participation in the study Confirmation that genetic testing was performed Encouragement to speak with a genetic counselor or one’s PCP about results Encouragement to share results with family members Contact information for research team Process elements for letter production Seek input of participants on letter design Prioritize creation of notification letters to improve readability Minimize reading difficulty for all materials (as reflected by Flesch Reading Ease, Flesch–Kincaid Grade Level or other measure) Consider new/novel formats (e.g., pictorial vignettes, Q&A format) to improve readability
